# Safety, Immunogenicity, and Mechanism of a Rotavirus mRNA-LNP Vaccine in Mice

**DOI:** 10.3390/v16020211

**Published:** 2024-01-31

**Authors:** Chenxing Lu, Yan Li, Rong Chen, Xiaoqing Hu, Qingmei Leng, Xiaopeng Song, Xiaochen Lin, Jun Ye, Jinlan Wang, Jinmei Li, Lida Yao, Xianqiong Tang, Xiangjun Kuang, Guangming Zhang, Maosheng Sun, Yan Zhou, Hongjun Li

**Affiliations:** Institute of Medical Biology, Chinese Academy of Medical Science & Peking Union Medical College, Yunnan Key Laboratory of Vaccine Research and Development on Severe Infectious Disease, Kunming 650118, China; luchenxing@student.pumc.edu.cn (C.L.); yjlz2314@163.com (Y.L.); chenrong@imbcams.com.cn (R.C.); huxiaoqing@imbcams.com.cn (X.H.); lqm212855240@163.com (Q.L.); igtheshy131@gmail.com (X.S.); linxiaochen@imbcams.com.cn (X.L.); yejun@imbcams.com.cn (J.Y.); lanlingyu@student.pumc.edu.cn (J.W.); lijinmei917@163.com (J.L.); adayao0926@163.com (L.Y.); tangxq8859@163.com (X.T.); kuangxiangjun@imbcams.com.cn (X.K.); zhangguangming@imbcams.com.cn (G.Z.); sunmaosheng@imbcams.com.cn (M.S.)

**Keywords:** rotavirus, mRNA vaccine, structural protein VP7, lipid nanoparticles, neutralizing antibody

## Abstract

Rotaviruses (RVs) are a major cause of diarrhea in young children worldwide. The currently available and licensed vaccines contain live attenuated RVs. Optimization of live attenuated RV vaccines or developing non-replicating RV (e.g., mRNA) vaccines is crucial for reducing the morbidity and mortality from RV infections. Herein, a nucleoside-modified mRNA vaccine encapsulated in lipid nanoparticles (LNP) and encoding the VP7 protein from the G1 type of RV was developed. The 5′ untranslated region of an isolated human RV was utilized for the mRNA vaccine. After undergoing quality inspection, the VP7-mRNA vaccine was injected by subcutaneous or intramuscular routes into mice. Mice received three injections in 21 d intervals. IgG antibodies, neutralizing antibodies, cellular immunity, and gene expression from peripheral blood mononuclear cells were evaluated. Significant differences in levels of IgG antibodies were not observed in groups with adjuvant but were observed in groups without adjuvant. The vaccine without adjuvant induced the highest antibody titers after intramuscular injection. The vaccine elicited a potent antiviral immune response characterized by antiviral clusters of differentiation CD8^+^ T cells. VP7-mRNA induced interferon-γ secretion to mediate cellular immune responses. Chemokine-mediated signaling pathways and immune response were activated by VP7-mRNA vaccine injection. The mRNA LNP vaccine will require testing for protective efficacy, and it is an option for preventing rotavirus infection.

## 1. Introduction

Rotaviruses (RVs) are classified as a genus in the family of Reoviridae. RVs are a major cause of diarrhea in young children worldwide [1]. Each year, infection by RVs results in ~114 million cases of acute gastroenteritis in children under 5 years of age. Diarrhea due to RV infection accounts for 5% of all global deaths in this age group, leading to ~200,000 infant fatalities [2,3]. Specific treatment is lacking, but vaccination is an effective means of preventing RV infection and the resulting gastroenteritis.

Seven live RV vaccines are in use: Rotarix (G1P [8]) [4]; RotaTeq (G1P [5], G2P [5], G3P [5], G4P [5], G6P [8]) [5]; Rotavac (G9P [11]) [6]; ROTASIIL (G1P [5], G2P [5], G3P [5], G4P [5], and G9P [5]) [7]; Lanzhou lamb rotavirus vaccine (G10P [12]) [8]; Rotalan (G2P [12], G3P [2], and G4P [12]) [9]; and Rotavin-M1 (G1P [8]) [7]. These vaccines have very important roles in reducing the burden of gastroenteritis caused by RV infection. These vaccines offer partial protection against infection, but their efficacy varies across different regions worldwide [10,11]. The risk of intussusception must also be considered [12]. Therefore, further optimization of live attenuated RV vaccines, or development of non-replicating RV vaccines to replace live attenuated RV vaccines (e.g., inactivated vaccines, recombinant subunit vaccines) is of great importance to further reduce the morbidity and mortality caused by RV infection.

Messenger (m)RNA vaccines are the third generation of nucleic acid vaccines after traditional (inactivated, live attenuated) vaccines and new (subunit, viral vector) vaccines. By introducing mRNA encoding one or more target antigenic proteins into the cytoplasm of host cells, antigenic proteins are expressed in host cells and then presented to the immune system of the host. This action activates the immune system to produce antibodies. This strategy has attracted extensive attention and research [13,14] due to its short development cycle, easy industrialization, simple and controllable production process, easy response to new variants, and better induction of humoral immunity and cellular immunity. Hence, vaccines based on the mRNA of viruses, bacteria, parasites, and tumor cells are being investigated [15].

In the early days of the COVID-19 outbreak, two mRNA vaccines of the novel coronavirus achieved great success, so mRNA vaccine technology has received widespread attention [16,17]. Research and development of mRNA vaccines are active worldwide, with the focus on treatment of infectious diseases and cancer [18]. In addition to severe acute respiratory syndrome coronavirus-2, mRNA technology is being used to create vaccines for influenza viruses [19], Zika virus [20], human immunodeficiency virus [21,22,23,24], respiratory syncytial virus [25], herpes simplex virus [26], varicella zoster virus [27], human cytomegalovirus [28], rabies virus [29], and Dengue virus [30]. A recent study showed that monovalent and trivalent LS-P2-VP8* induced superior humoral responses to P2-VP8* in guinea pigs, with encouraging responses detected against the most prevalent P genotypes [31].

RV is an unenveloped double-stranded RNA virus. The genome comprises 11 segmented double-stranded RNAs that encode six structural proteins (VP1–VP7) and six non-structural proteins (NSP1–NSP6) [32,33,34]. The VP7 protein, encoded by the structural gene VP7, along with the structural protein VP4, constitutes the outermost layer of the RV structure. VP7 and VP4, two capsid proteins, harbor neutralizing epitopes and have vital roles in invading and infecting target cells [35]. Consequently, they are utilized frequently as candidates for genetically engineered RV vaccines [36]. Glycoprotein VP7 is a structurally neutralizing antigen of RVs that can elicit the production of immunoglobulin (Ig)G antibodies, which are associated with protection afforded by the immune system [37].

Herein, we assessed the immunogenicity of an mRNA vaccine for RVs. Our approach involved creating an mRNA vaccine with an encoded G1P [8] RV VP7 protein and enveloping it in lipid nanoparticles (LNP). Subsequently, mice were immunized with doses (2, 5, or 10 μg) through intramuscular (IM) or subcutaneous (SC) routes. Finally, humoral and cellular immunity were assessed following three immunizations. The VP7 mRNA vaccine could elicit production of RV-specific antibodies and activate T-cell immune responses. The mRNA LNP vaccine will require testing for protective efficacy.

## 2. Materials and Methods

### 2.1. Ethical Approval of the Study Protocol

The experimental protocol was approved (DWLL202208007) by the Experimental Animal Welfare Ethics Committee of the Institute of Medical Biology within the Chinese Academy of Medical Sciences (Beijing, China). Bodyweight and temperature were monitored daily. Animals exhibiting significant reductions in these parameters (as well as other severe health issues) were killed humanely to enable sample collection.

### 2.2. Cells and Viruses

The virus named “ZTR-68-A” (G1P [8]) was obtained from a child suffering from diarrhea in Yunnan Province (China). ZTR-68-A (G1P [8]) was preserved by the Molecular Biology Laboratory in the Institute of Medical Biology within the Chinese Academy of Medical Sciences. HEK293 cells were obtained from OBIO Technology (Shanghai, China). MA104 cells were stored in the Laboratory of Molecular Biology, Institute of Medical Biology, Chinese Academy of Medical Sciences (Kunming, China).

### 2.3. Generation of mRNA and mRNA-LNP

Wild-type rotaviral VP7 (GenBank: JX509940.1) was synthesized by Integrated DNA Technologies (Coralville, IA, USA) and constructed in the pUC57-Kan-SapI-free vector by Genscript Biotech (Piscataway, NJ, USA). The construct contained a T7 promoter site for in vitro transcription of mRNA, a 5′ untranslated region (UTR) derived from rotavirus, a full-length sequence of VP7 CDS, a 3′UTR derived from human β-globin [38,39], and a poly A tail with 115 nucleosides. The plasmid was extracted and linearized using BspQI enzyme (DD4302; Vazyme, Nanjing, China). After completion, DNA magnetic beads (N411; Vazyme) were used for purification. RNA was amplified using the T7 High Yield RNA Transcription Kit (N¹-Me-Pseudo UTP) (DD4202; Vazyme). RNA magnetic beads (N412; Vazyme) were used to purify IVT production. Then, the mRNA was capped with Vaccinia Capping Enzyme (DD4109; Vazyme) and 2’-O-Methyltransferase (DD4110; Vazyme). Subsequently, mRNA was purified using RNA magnetic beads (N412; Vazyme) and dissolved in RNase-free water. The mRNA concentration was determined using an ultra-micro-spectrophotometer (Thermo Fisher Technologies, Waltham, MA, USA). After purification, mRNA was stored at −80 °C until use.

After determining the expression effect of VP7, the capped and purified mRNA was diluted with 50 mmol/L sodium acetate buffer (pH5.5) to 200 ng/µL, and then LNP-wrapped. LNP’s components can be divided into ionizable lipid, DSPC, cholesterol and polyethylene glycol-lipid (AVT, Shanghai, China). The preparation method is to dissolve the above four lipid components in anhydrous ethanol according to a molar ratio of 50:10:38.5:1.5 and then form LNP after blowing and mixing. mRNA-LNP was obtained using a microfluidic mixer (INano™L; Apenzy Biosciences, Shrewsbury, MA, USA) to complete the process, and the flow rate ratio of LNP and mRNA was 1:3. Meanwhile, empty LNP was also included as a control. The obtained mRNA-LNP vaccine was diluted 100 times with Tris-HCL buffer (PH7.5), and then the vaccine was concentrated to the original volume using an ultrafiltration tube (100 K) to complete the replacement of anhydrous ethanol. Finally, the preparation was passed through a 0.22 µm filter and stored at 4 °C until use. The size and potential of LNP were analyzed with a laser particle-size analyzer (Malvern Instruments, Malvern, UK). A RiboGreen^®^ assay (Thermo Fisher Technologies) was employed to determine the encapsulation and concentration of mRNA. Transmission electron microscopy (JEM-1200EX, JEOL, Tokyo, Japan) was used to observe the morphology and size of LNP.

### 2.4. Transfection and Viral Protein Expression

mRNA was transfected into HEK293 cells using a transfection reagent (jetMESSENGER™; PolyPlus, Brant, France). After 24 h, total cell protein and whole-cell supernatants were collected. Each lysate sample underwent sodium dodecyl sulfate–polyacrylamide gel electrophoresis on 10% gels using TRIS-HCl. Then, proteins were transferred onto polyvinylidene fluoride (PVDF) membranes, which were enclosed in TBST (Tris-buffered saline containing Tween 20) solution with 5% skimmed milk. PVDF membranes were treated with primary (rabbit anti-VP7) antibody, followed by addition of a secondary antibody (horseradish peroxidase-coupled goat anti-rabbit IgG; Abcam, Cambridge, UK), followed by incubation for 1 h at room temperature. After washing, treated PVDF membranes were exposed to a highly sensitive luminescence solution (PK10003; Proteintech, Chicago, IL, USA) and imaged using an electrochemiluminescence system (Thermo Fisher Scientific, Waltham, MA, USA).

### 2.5. Mouse Experiments

**Figure 1:**
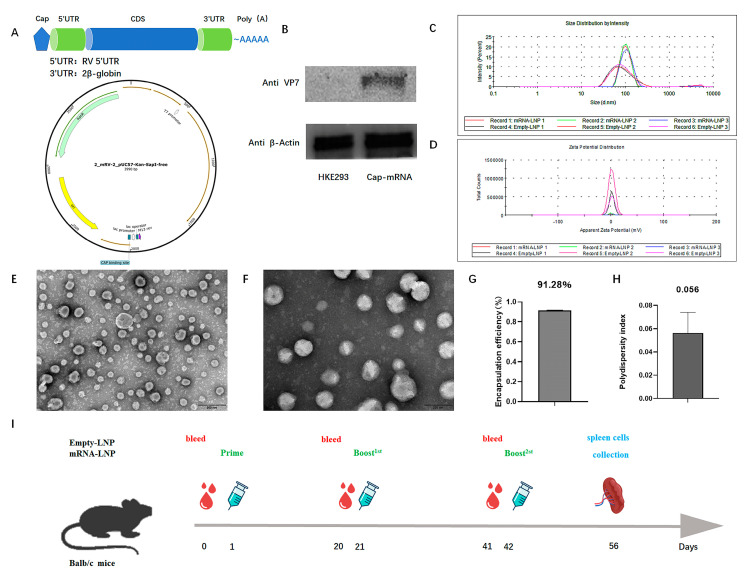
Vaccine characterization and immunization strategy. (**A**) Model of vaccine construction. (**B**) Detection of mRNA expression after transfection of Cap-mRNA to HEK293 cells by Western blotting; β-Actin was used as an internal reference. (**C**) Particle size detection with a Malvin laser granmeter. (**D**) Electric potential of the mRNA-LNP. (**E**,**F**) Shape of mRNA-LNP particles observed by transmission electron microscopy. The scale of (**E**,**F**) are 500 nm and 200 nm, respectively. (**G**) Percentage encapsulation of the encapsulated vaccine. Data are represented as mean ± SD. (**H**) PDI of the encapsulated vaccine. Data are represented as mean ± SD. (**I**) Schedule of immunization with the VP7-mRNA vaccine and blood collection. Mice were divided into three groups: IM injection with adjuvant; SC injection with adjuvant; and IM injection without adjuvant. Each group had a dosage subgroup of 2, 5 and 10 μg. The immunization procedure was three doses with an interval of 21 days, and the spleen was removed 14 days after the final immunization.

Vaccines were mixed with/without an equal volume of aluminum hydroxide. Then, vaccines were injected in female Balb/c mice aged 6–8 weeks. Mice were divided into three groups: IM injection with adjuvant (group A); SC injection with adjuvant (group B); and IM injection without adjuvant (group C). Each group had dosage subgroups of 2, 5 and 10 μg. Subgroups were named with their group number–injection method–dose. In the three vaccine groups, sera were collected at days 0, 20, 41, and 56 to evaluate the immunogenicity of the vaccine. The immunization scheme is shown in Figure 1.

### 2.6. Detection of IgG Antibody and Neutralizing Antibody

For IgG antibodies’ detection, the RV concentrate was added to an enzyme-linked immunosorbent assay-coated solution (C1050; Solarbio, Beijing, China) at a ratio of 1:100. Next, the mixture was coated onto a 96-well plate (Corning, NY, USA) with a flat bottom at a volume of 100 µL per well. The plate was kept overnight at 4 °C and subsequently washed and sealed with 3% bovine serum albumin for 1 h. Serum samples were diluted in buffer and incubated for 1 h at 37 °C, followed by five washings. Horseradish peroxidase-conjugated goat anti-mouse IgG antibody (HA1006; Huabio, Woburn, MA, USA) was diluted in 3% BSA at 1:20,000 and incubated for 1 h. The plates were evaluated using an EPOCH microplate reader (BioTek, Winooski, VT, USA) at an absorbance of 450 with a reference wavelength of 650 nm. If the A450 values of the serum dilution were higher than 0.105, the IgG/IgA antibody was considered to be positive, while the reciprocal of the highest positive serum dilution was considered as the IgG/IgA titer.

To detect neutralizing antibodies, sera with different dilutions were mixed with RV and incubated at 37 °C for 2 h. The mixture was then added to a 96-well plate filled with MA104 cells and incubated at 37 °C for 7 days. After freezing and thawing twice, the lysate was transferred to a 96-well plate coated with RV antibody, incubated at 37 °C for 1 h, then washed with PBST 5 times and added RV enzyme labeled antibody at 1:3000 dilution. Absorbance was measured by Biotek at 450 nm and 650 nm using an enzyme-labeling instrument (Biotek).

### 2.7. Flow Cytometry

The whole blood of mice was collected using collection vessels coated with anticoagulant (heparin sodium). Then, surface markers were stained with CD3ε-PerCP, CD4-APC, and CD8-PE (Biolegend, San Diego, CA, USA) for 30 min. Next, red blood cell lysate (R1010; Solarbio) at 3 × volume was added, followed by gentle vortex-mixing or tube inversion. After cooling on ice for 15 min, centrifugation (450× *g*, 10 min, 4 °C) was undertaken. The supernatant was discarded and red blood cell lysate (2 × volume) was added. The mixture was agitated gently and centrifuged (450× *g*; 10 min, 4 °C). The supernatant was discarded and the cell pellet resuspended with 500 µL cell-staining buffer (420201; Darco, Syracuse, NY, USA) for analyses on a high-speed flow cytometer (BD LSRFortessa™; BD Biosciences, Franklin Lakes, NJ, USA). The resulting data were analyzed using FlowJo V10 (BD Biosciences).

Mouse spleens were isolated and ground following strict aseptic procedures. Splenic lymphocytes were isolated using Mouse Lymphocyte Isolation Solution (7211011; Darko, Bedford Heights, OH, USA) and fixed with Cyto-Fast Fix/Perm Buffer (426803; Darko) and CD3ε-PerCP. Surface markers were stained for 30 min using CD4-APC and CD8-PE (BioLegend). After centrifuging for 350× *g*, 5 min, 500 µL of cell-staining buffer (420201; Darco) was added, and the sample was analyzed on a high-speed flow cytometer (BD LSRFortessa). FlowJo V10 was used for data analyses.

### 2.8. Enzyme-Linked Immunosorbent Spot (ELISpot)

Spleen lymphocytes (2 million cells/well from immunized mice) were cultured in 96-well plates for measurement of IFN-γ expression using an ELISpot assay kit (3321-4AST-2; Mabtech, Stockholm, Sweden) following the manufacturer’s instructions. A VP7 peptide (final concentration = 20 μg/mL) was utilized to stimulate specific T-cell responses. An identical volume of PMA + ionomycin was utilized as a positive control. Spots were enumerated using an ELISpot reader system (Autoimmun Diagnostika, Strasbourg, France).

### 2.9. Detection of Cytokines in Serum

After the slide chip had dried completely, the cytokine standard was prepared. Sample diluent (100 μL) was added to each hole of the chip. The quantitative antibody chip was incubated on a shaker for 1 h at room temperature before being closed. After cleaning, a detection antibody was added to each well followed by incubation overnight on a shaker for 2 h at 4 °C. After cleaning, CY3-streptaavin was added to each well and the slide wrapped in aluminum foil and incubated on a shaker for 1 h at room temperature. After additional cleaning, fluorescence detection was undertaken using a laser scanner (InnoScan 300 Microarray Scanner; Innopsys, Chicago, IL, USA). Data analyses were carried out using QAM-CYT-1 (Raybiotech, Norcross, GA, USA).

### 2.10. Transcriptome Sequencing of Peripheral Blood Mononuclear Cells (PBMCs)

Samples of PBMC from two groups of mice (mRNA-LNP-immunized and control) were collected 14 days after the third immunization. Total RNA was extracted using TRIzol^®^ Reagent (Invitrogen, Carlsbad, CA, USA) according to the manufacturer’s protocols. Then, mRNA libraries were constructed using the VAHTS Universal V6 RNAseq Library Prep Kit according to manufacturer’s (Vazyme) instructions. Sequencing and analyses of the transcriptome were conducted by OE Biotech (Shanghai, China). Raw reads in fastq format were processed using fastp’ (https://github.com/OpenGene/fastp, accessed on 8 April 2020). Low-quality reads were removed to obtain clean reads. Then, ~6.97 million clean reads for each sample were retained for subsequent analyses. Clean reads were mapped to the reference genome using HISAT22 (https://github.com/DaehwanKimLab/hisat2, accessed on 8 June 2017). The fragments per kilobase million (FPKM)3 of each gene was calculated. The read counts of each gene were obtained by HTSeq-count4 (https://github.com/htseq/htseq/blob/main/doc/htseqcount.rst, accessed on 8 October 2023). Analyses of differential expression were undertaken using DESeq25 (https://github.com/thelovelab/DESeq2, accessed on 8 October 2023). Q < 0.05 and fold change (FC) > 1.5 or <0.67 were set as thresholds for significantly differentially expressed genes (DEGs). Based on the hypergeometric distribution, enrichment analyses of DEGs were carried out based on the Gene Ontology (GO; https://geneontology.org, accessed on 8 October 2023), Kyoto Encyclopedia of Genes and Genomes (KEGG; www.genome.jp accessed on 8 October 2023), Reactome (https://reactome.org, accessed on 8 October 2023), and WikiPathways (https://www.wikipathways.org, accessed on 8 October 2023) databases using R 3.2.0 Institute for Statistical Computing (Vienna, Austria).

### 2.11. Statistical Analyses

Prism 9.0.2.161 (GraphPad, La Jolla, CA, USA) was used for data analyses and mapping. Experimental results are expressed as the geometric mean ± standard error. Between-group differences were analyzed using a two-tailed Student’s *t*-test or Tukey’s multiple comparison test. *p* < 0.05 was considered significant.

## 3. Results

### 3.1. Construction, Characterization, and Protein Expression of RV VP7 mRNA-LNP

The plasmid was synthesized according to the design strategy for the mRNA vaccine, as shown in Figure 1A. The capped products were transfected with 2 μg of vaccine into HEK293 cells, and mRNA expression was analyzed at the cellular level via Western blotting. The resulting Cap-mRNA was expressed in cells (Figure 1B). After the Cap-RNA had been encapsulated with LNP using microfluidic technology, the particle size and potential were measured and observed under an electron microscope. The particle size of the obtained LNP-mRNA was ~100 nm (Figure 1C) and the electric potential was ~0 mV (Figure 1D). The product demonstrated spherical particles with round edges according to TEM (Figure 1E,F). The percent encapsulation of the vaccine was 91.28% according to the RiboGreen kit (Figure 1G). Furthermore, the average polydispersity index was < 0.105 (Figure 1H).

### 3.2. RV mRNA Vaccine Elicited Effective Humoral and Cellular Immune Responses

**Figure 2:**
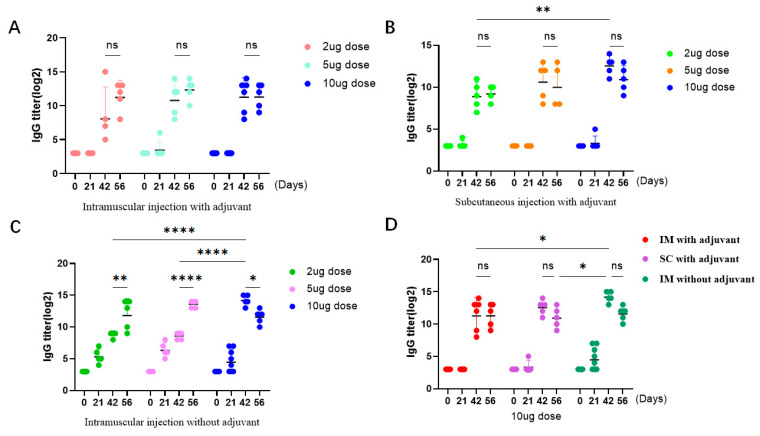
The vaccine triggered humoral immune responses in mice. (**A**) Levels of IgG antibodies that the vaccine triggers in 2, 5, and 10 μg groups after intramuscular injection with adjuvant. (**B**) Levels of IgG antibodies that the vaccine triggers in 2, 5, and 10 μg groups after subcutaneous injection with adjuvant. (**C**) Levels of IgG antibodies that the vaccine triggers in 2, 5, and 10 μg groups by intramuscular injection without adjuvant. (**D**) The effects of the three immunization routes were compared in the 10 μg dose group. Data are presented as geometric mean with geometric SD. Significant differences were determined by a two-way ANOVA (* *p* < 0.05, ** *p* < 0.01 and **** *p* < 0.0001; ns. indicates not significant).

The serum level of IgG antibody in mice was measured to evaluate the immunogenicity of the VP7-mRNA vaccine. Sera from mice were collected at days 0, 20, 41, and 56 for measurement of IgG antibody for the three-dose group. Sera from mice were collected at days 0, 20, and 35 for measurement of IgG antibody for the two-dose group. A four-fold increase in the serum IgG antibody titer induced by doses of 2, 5, and 10 μg indicated that the conversion was 100%. After two immunizations, in group A, the IgG antibody titer (log_2_) (GMT) increased in the 2, 5, and 10 μg groups by 8.05, 10.77, and 11.27 (Figure 2A), whereas in group B it increased by 8.89, 10.62, and 12.56 (Figure 2B), respectively. In group C, the IgG antibody titer increased by 8.79, 8.59, and 14.18 (Figure 2C). After three immunizations, in group A, the IgG antibody titer (log_2_) (GMT) increased in the 2, 5, and 10 μg groups by 11.23, 12.32, and 11.28; in group B, it increased by 9.21, 10, and 10.91; and and in group C, it increased by 11.8, 13.59, and 11.55, respectively (Figure 2A,C). After comparing the three immunization routes, the IgG antibody level was highest in the intramuscular injection group without adjuvant. There was no statistically significant difference in the IgG antibody level between the two-dose and three-dose immunization groups with adjuvant, while there was a statistically significant difference in the two-dose and three-dose immunization groups without adjuvant (Figure 2D).

**Figure 3:**
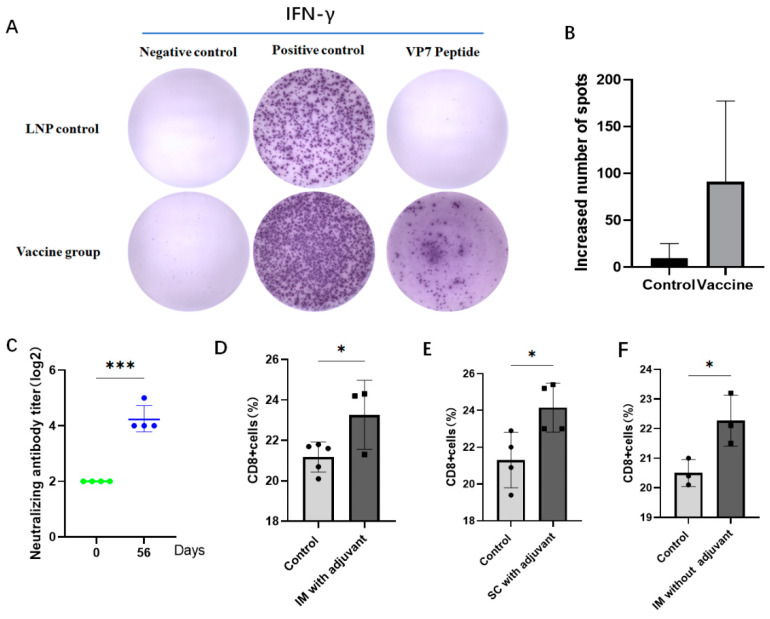
The vaccine triggered cellular immunity and neutralizing antibodies in mice. (**A**) In the group of 5 µg by intramuscular injection without adjuvant, the amount of interferon-γ produced by splenic lymphocytes in the control group and vaccine group was measured by ELISpot. (**B**) The number of spots in the control group and vaccine group was mapped and counted in ELISpot. Data are represented as mean ± SD. (**C**) In the group of 10 µg by intramuscular injection without adjuvant, the vaccine triggered neutralizing antibodies after the third immunization. Data are presented as geometric mean with geometric SD. Significant differences were determined by an unpaired *t* test. (*** *p* < 0.001). (**D**) The percentage of CD8^+^ cells in total spleen lymphocytes in the group of 10 µg by intramuscular injection with adjuvant. (**E**) The percentage of CD8^+^ cells in total spleen lymphocytes in the group of 10 µg by subcutaneous injection with adjuvant and (**F**) the group of 10µg by intramuscular injection without adjuvant. Data are presented as mean ± SD. Significant differences were determined by an unpaired *t* test (* *p* < 0.05).

To explore if the VP7-mRNA vaccine could induce a cellular immune response after immunization of mice, splenic lymphocytes were collected for flow cytometry and ELISpot detection. In the group of 5 µg by intramuscular injection without adjuvant, VP7-specific IFN-γ responses could be elicited according to the ELISpot assay (Figure 3A). A statistical chart of the number of spots is displayed (Figure 3B). Hence, in the group with 5 µg by intramuscular injection without adjuvant, the vaccine stimulated a T helper (Th)1 cell immune response in mouse lymphocytes. In the group of 10 µg by intramuscular injection without adjuvant, after the third injection, the neutralizing antibody titer (log_2_) (GMT) increased by 4.23 (Figure 3C). For the 10 µg group, flow cytometry revealed that the percentage of CD8^+^ cells increased after vaccine immunization of mice, which suggested an increase in the number of CD8^+^ T cells (Figure 3D–F).

### 3.3. Transcriptome Sequencing of Peripheral Blood Mononuclear Cells (PBMCs)

**Figure 4:**
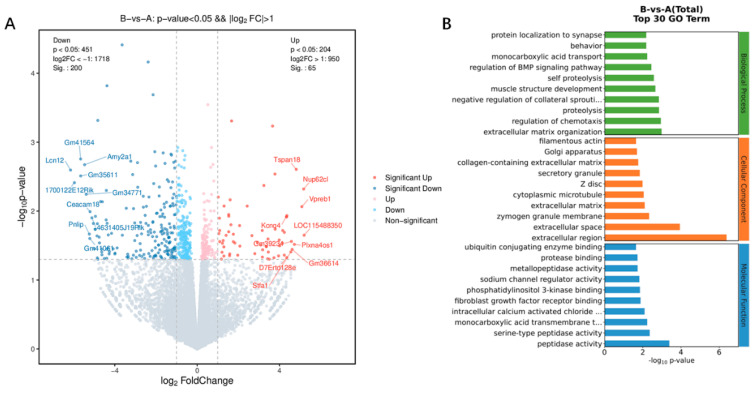
Changes in gene expression of PBMCs. (**A**) The differences generated by the comparison are reflected in the volcano map. Gray shows genes with a non-significant difference in expression. Red and blue are genes with a significant difference in expression. The horizontal axis is log_2_ fold change. The vertical axis is −log_10_ of the *p*-value. (**B**) Analyses of functional enrichment (using the GO database) of the top 30 genes (based on selection of GO items corresponding to PopHits ≥ 5 in the three categories and ranking 10 items from largest to smallest according to the corresponding −log_10_ *p*-value of each item).

To investigate the gene expression changes in PBMCs after vaccine injection, transcriptome sequencing on the splenocytes of mice that received 10 µg of the vaccine without adjuvant was performed. Transcriptome sequencing was carried out 14 days after vaccination. Then, DEGs were identified through FC. The threshold set for genes with upregulated and downregulated expression was FC ≥ 2.0. Compared with the genes in the control group, 65 genes had upregulated expression and 200 genes had downregulated expression after one immunization. The top 10 genes with upregulated expression were *LOC115488350*, *Nup62cl*, *Vpreb1*, *Tspan18*, *Plxna4os1*, *Gm36614*, *Gm39234*, *Stfa1*, and *Kcnq4*. The top 10 genes with downregulated expression were *Lcn12*, *1700122E12Rik*, *Gm35611*, *Gm41061*, *Amy2a1*, *Gm34771*, *Pnlip*, *Gm41061*, *Ceacam18*, and *4631405J19Rik*. Among these DEGs, *LOC115488350* had the highest upregulation after one dose of vaccine, and *Lcn12* had the highest downregulation (Figure 4A).

**Figure 5:**
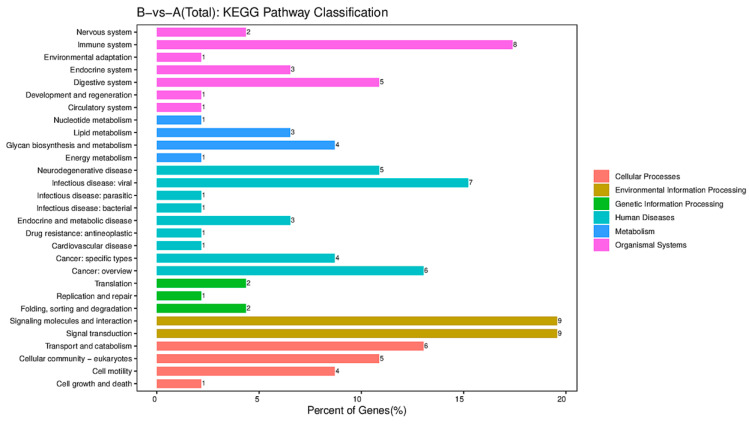
KEGG pathway classification. The horizontal axis is the percentage of the total number of genes annotated to each pathway (differentially expressed genes (DEGs)) and all genes annotated to the KEGG database (DEGs). The vertical axis represents the pathway name, and the number on the right side of the column represents the number of DEGs annotated to this pathway.

The DEGs after each immunization were subjected to analyses of functional enrichment using the GO database based on biological process (BP), cellular component (CC), and molecular function (MF). For BP, the DEGs were enriched mainly in “extracellular matrix organization”, whereas they were enrieched mainly in “extracellular space” in CC, and “peptidase activity” in MF (Figure 4B). Enrichment of the signaling pathways of DEGs was assessed using the KEGG database. DEGs showed enrichment in “immune system”, “infectious disease: viral”, “signaling molecules and interaction”, and “signal transduction”. The “immune system” pathway was clustered in four terms: “chemokine signaling pathway”, “complement and coagulation”, “NOD-like receptor signaling pathway” and “intestinal immune network for IgA production”. *C-C motif chemokine 28* (*CCL28*; FC = 2.9), *serpin f2* (FC = 10.3), and *caspase 12* (FC = 3.0) were involved in the “immune system” pathway. The “infectious disease: viral” pathway was clustered in three terms: “hepatitis B”, “human cytomegalovirus infection” and “Kaposi sarcoma-associated herpes virus infection”. *Caspase 12* (FC = 3.0), platelet-derived growth factor receptor a (*Pdgfra*) (FC = 9.5), and *Cd200r2* (FC = 2.9) were involved in the “infectious disease: viral” pathway (Figure 5).

### 3.4. Vaccine Safety

**Figure 6:**
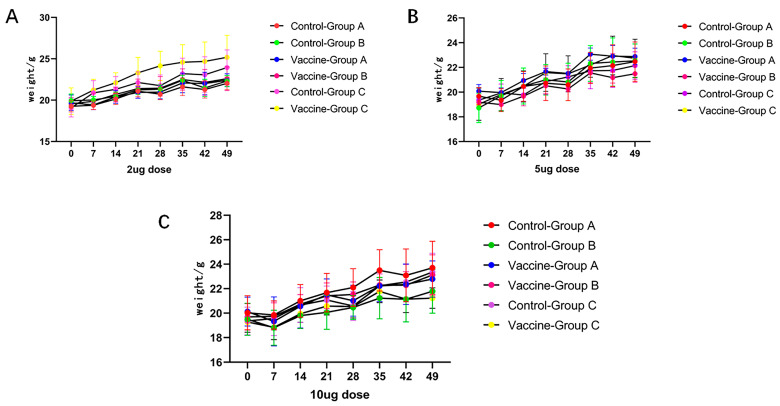
Changes in the body weight of mice. Starting at week 0, weight was measured weekly until the end of week 7. The weight of mice in different dose groups was divided into a (**A**) 2 μg group, (**B**) 5 μg group, and (**C**) 10 μg group. The weight data are presented in the form of mean ± SD.

To evaluate the safety of the vaccine, body weight monitoring of mice was performed after immunization. The weight of the mice was measured once a week. The body weight of mice in each group did not decrease significantly, and maintained a stable increase (Figure 6). The results showed that the mice grew normally during immunization. Immunizing mice with the vaccine did not affect body weight, indicating that the vaccine had no serious side effects.

## 4. Discussion

mRNA technology has been utilized to develop vaccines for different infectious diseases. The first mRNA vaccine was created for influenza viruses [40]. Moderna completed phase I clinical trials using LNP as a carrier for the influenza vaccines H10N8 and H7N9. This mRNA vaccine prevented and protected against influenza A infection while exhibiting strong immunogenicity, safety, and tolerance in humans [41]. Meyer and colleagues administered two mRNA vaccines that encode Ebola virus (EBOV) glycoproteins to guinea pigs infected with EBOV. Those guinea pigs could produce EBOV glycoprotein-specific IgG antibodies and EBOV-neutralizing antibodies. These phenomena resulted in all the guinea pigs infected with EBOV surviving [42]. Roth et al. developed an LNP-modified mRNA vaccine that encoded the non-structural protein of Dengue virus type I as the target antigen. Then, they immunized mice with human leukocyte antigen class-I molecules. Virus-specific CD8^+^ T cells (which have a crucial protective role) [43] were generated.

RV infection is a significant contributor to diarrhea among infants and young children worldwide. It accounts for 5% of deaths among children under 5 years of age [2,44]. Live attenuated RV vaccines can prevent RV infection to a certain extent, but are hampered by safety issues (e.g., intestinal adverse reactions), efficacy differences between countries (developed countries have higher efficacy than developing countries), and expenses (e.g., problems in cold-chain transportation). Development of RV vaccines faces significant challenges [45,46,47,48,49]. To further reduce the risk of the morbidity and mortality caused by RV infection, development of a new RV (e.g., mRNA) vaccine is imperative because it could supplement the use of live vaccines.

The VP7 protein serves as the structural protein for, and determines the G type of, RVs. It plays a crucial part in the infection process of RV cells and in the assembly of RV particles. It also contains several neutralizing epitopes, making it a key neutralizing antigen for RVs [1]. We developed and formulated a VP7-mRNA vaccine expressing the UTR of RVs and investigated its immunogenicity in terms of dose response and immune pathway. Immunization with the VP7-mRNA vaccine (2, 5, or 10 µg) induced a humoral immune response and cytotoxic T-cell response. Furthermore, adjuvant-free vaccination with 10 µg of the vaccine resulted in higher IgG antibody levels. The group of mice that did not receive adjuvant consistently outperformed the group receiving adjuvant. Non-replicating vaccines primarily activate the innate immune response at the inoculation site, which highlights the importance of the site and route of inoculation. In the present study, IM injection of the VP7-mRNA vaccine produced greater stimulation of humoral immunity than that by SC injection. This effect occurred because of the abundance of antigen-presenting cells (APCs) in muscle blood vessels. APCs can trigger the immune response rapidly after capturing the antigen, whereas adipose tissue contains fewer APCs, thereby making IM injection more effective than SC injection. Furthermore, it has been reported that IM injection of mRNA-LNP is safer than cortical or intravenous injection for inducing the production of anti-LNP antibodies. Our ELISpot results indicated that interferon-γ could be stimulated by the VP7-mRNA vaccine. This vaccine immunization could also stimulate the proliferation of activated T cells, activate CD4+ T cells to differentiate into Th0 cells, promote the differentiation of Th0 cells to Th1 cells, and secrete interferon-γ. The amount of neutralizing antibody is relatively low. In the future, the VP7 sequence and structural optimization are needed to facilitate the production of more highly neutralizing antibodies.

The total RNA of PBMC samples was extracted for transcriptome sequencing to explore the gene expression changes in PBMCs after vaccine injection. Among the top 10 DEGs, the protein encoded by *Vpreb1* belongs to the Ig superfamily and is expressed selectively at the early stages of B-cell development in pro-B and early pre-B cells. This gene encodes the iota polypeptide chain that is associated with the µ chain of the Ig molecule to form a molecular complex which is expressed on the surface of pre-B cells. This molecular complex is thought to regulate *Ig* rearrangements in the early steps of B-cell differentiation. Immunization by the vaccine can cause changes in levels of chemokines and immune regulation-related genes. Among them, *CCL28* (FC = 2.9) and *Pdgfra* (FC = 9.5) have important regulatory roles in triggered immune system- and infectious disease-related pathways. CCL28 is a β or CC chemokine. The chemokine encoded by this gene displays chemotactic activity for resting CD4 T cells or CD8 T cells and eosinophils and IgA production of the intestinal immune network. *Pdgfra* acts as a cell surface receptor for platelet-derived growth factor (PDGF)A, PDGFB, and PDGFC, and has an essential role in the regulation of embryonic development, cell proliferation, cell survival, and chemotaxis.

## 5. Conclusions

In conclusion, we designed a novel VP7-mRNA vaccine carrying the viral UTR-encoded VP7 protein of RV. The mRNA vaccine could express the rotaviral VP7 protein and induce humoral and cellular immunity by regulating immune system-related genes and multiple signaling pathways. It is feasible to use VP7 as an immunogen for rotavirus mRNA design. The protective efficacy of the novel RV candidate vaccine should be determined before further development. In addition, multivalent and multi-target vaccines also should be considered.

## Data Availability

Data are contained within the article.
